# Dissociation between area TE and rhinal cortex in accuracy vs. speed of visual categorization in rhesus monkeys

**DOI:** 10.3389/fnbeh.2024.1481478

**Published:** 2024-11-21

**Authors:** Bing Li, Kaleb Lowe, Samarth Chandra, Gang Chen, Mark A. G. Eldridge, Barry J. Richmond

**Affiliations:** ^1^Laboratory of Neuropsychology, National Institute of Mental Health, National Institutes of Health, Bethesda, MD, United States; ^2^Scientific and Statistical Computing Core, National Institute of Mental Health, National Institutes of Health, Bethesda, MD, United States

**Keywords:** visual categorization, area TE, rhinal cortex, rhesus monkey, decision-making

## Abstract

In real-world vision, objects may appear for a short period, such as in conjunction with visual search. Presumably, this puts a premium on rapid categorization. We designed a visual categorization task cued by briefly presented images to study how visual categorization is processed in an ethologically relevant context. We compared the performance of monkeys with bilateral area TE lesions, and those with bilateral rhinal cortex lesions, to control animals. TE lesions impaired the accuracy but not the speed of visual categorization. In contrast, rhinal cortex lesions did not affect the accuracy but reduced the speed of visual categorization. A generalized drift-diffusion model (GDDM) with collapsing bounds was fitted to the data. The drift rate was equivalent across all groups, but the decision bounds collapsed more slowly in the rhinal group than in the other two groups. This suggests that, although evidence is accumulated at the same rate in all groups, the rhinal lesion results in slower decision-making.

## 1 Introduction

In the real world, visual objects often appear only briefly as they pass through our field of view. Rapid categorization of those objects is essential to guide the correct response, e.g., run from a source of danger or approach a rewarding object. Visual categorization requires integrating complex features, generating a template for each category and/or perceptual boundary between different categories, and assigning an object to one of those categories. The inferotemporal cortex (IT), comprising areas TEO and TE, plays a crucial role in object recognition and visual categorization (Kobatake and Tanaka, 1994; Tanaka, 1996; Grill-Spector and Weiner, 2014; Conway, 2018). A brain area adjacent to IT, the rhinal cortex, is known to support the valuation of visual objects (Liu et al., 2000; Mogami and Tanaka, 2006; Ohyama et al., 2012), object recognition memory (Meunier et al., 1993; Buckley et al., 2001; Bussey et al., 2003), and association among visual objects (Murray et al., 1993; Higuchi and Miyashita, 1996; Fujimichi et al., 2010).

In the present study, we designed a visual categorization task cued by briefly presented images of visual objects to study the roles of area TE and rhinal cortex in rapid visual categorization. Three groups of monkeys—unoperated controls, those with bilateral area TE lesions, and those with bilateral rhinal cortex lesions—categorized morphed images of cats and dogs presented for 25, 50, 100, 250, or 500 ms, in an interleaved fashion. Accuracy and processing time were taken as dependent measures. A generalized drift-diffusion model was used to model the animals’ decision-making process.

## 2 Methods

### 2.1 Subjects

Nine male monkeys (*Macaca mulatta*) participated in this study. Three (5–6 years old, weighing 6.9–9.0 kg) received bilateral aspiration removals of area TE 2 years before the experiments in the present study (Matsumoto et al., 2016; Eldridge et al., 2018). Three (7 years old, weighing 7.0–14.5 kg) received bilateral aspiration removals of the rhinal cortex 3 years before the experiments in the present study (Eldridge et al., 2018). Three (8–11 years old, weighing 7.8–9.5 kg) were unoperated controls. The training histories of the groups were very similar; in particular, they had all undergone the same order of training in object recognition and categorization tasks immediately prior to this study (Eldridge et al., 2018). All experimental procedures followed the guidelines of the *Institute of Medicine Guide for the Care and Use of Laboratory Animals* and the regulations from the ILAR Guide for the Care and Use of Laboratory Animals and were performed under an Animal Study Proposal approved by the Animal Care and Use Committee of the National Institute of Mental Health.

### 2.2 Two-interval forced choice task

**Figure 1:**
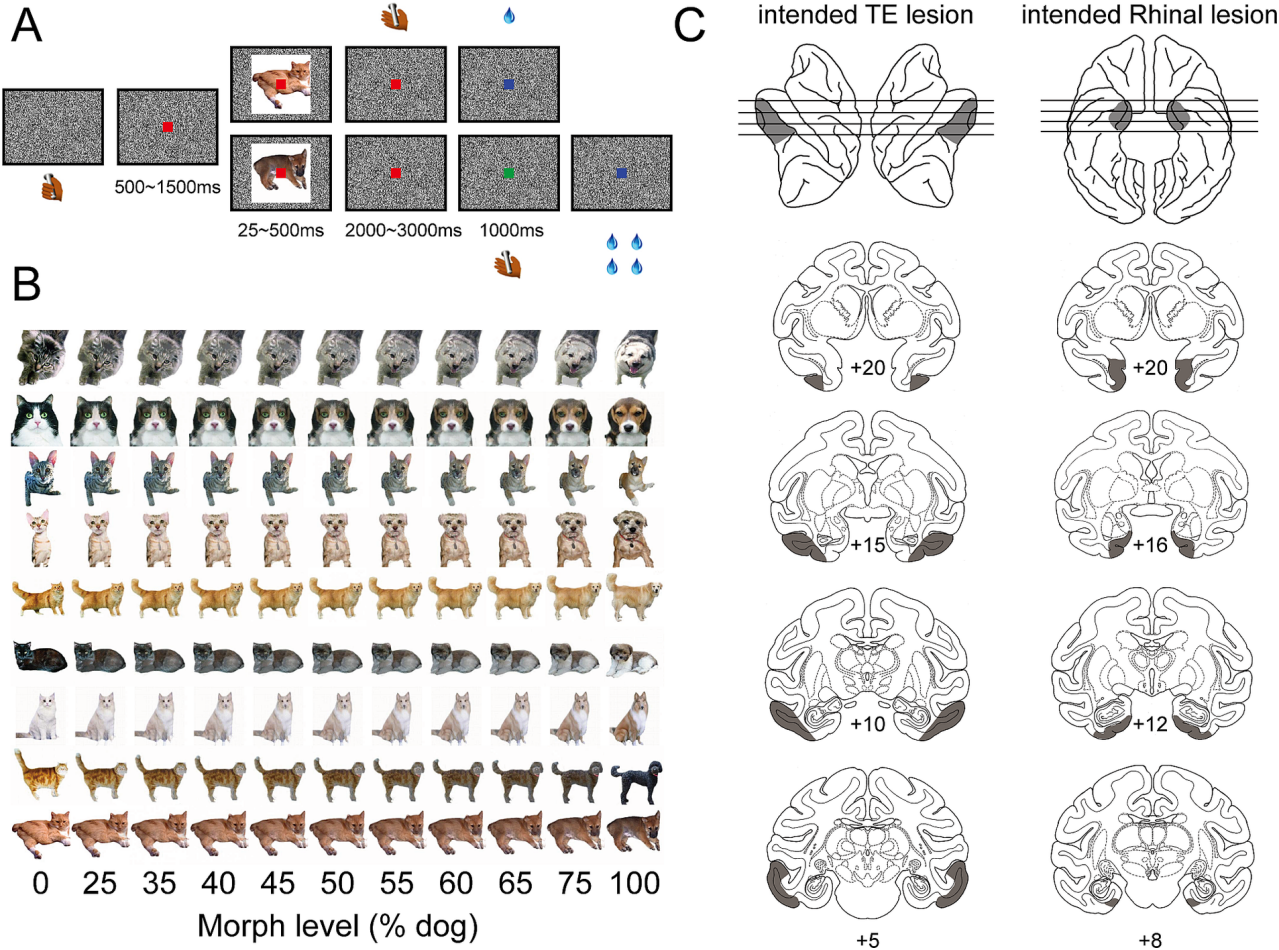
Behavior task and intended lesion areas. **(A)** Two-interval forced-choice task. Cue images appeared briefly, 25, 50, 100, 250, or 500 ms, in each trial. Monkey was required to release the bar during the red-dot interval for a more cat-like image and during the green-dot interval for a more dog-like image. **(B)** Cue image set. Each row shows one series of cue images which were morphed between one cat and one dog image with different ratios—a higher proportion of dog images from the left to the right. **(C)** Intended lesion areas. The shaded area on the left column shows the intended lesion area in area TE—bilaterally, from anterior to posterior, on the sagittal view of left and right hemispheres (top row) and coronal sections (lower rows). The shaded area on the right column shows the intended lesion area in the rhinal cortex—bilaterally, from anterior to posterior, on the ventral view of the left and right hemispheres (top row) and coronal sections (lower rows).

A trial began when a monkey held the touch bar (Figure 1A). A red dot appeared on the center of the screen. 500 ms to 1,500 ms later, a cue image, morphed to be either more cat-like or more dog-like (Figure 1B), was presented for a limited period of time (randomly selected from 25, 50, 100, 250, or 500 ms). The red dot turned green 2000 to 3,000 ms after the cue image was removed. The green dot disappeared after 1,000 ms. The monkey needed to release the touch bar in one of two intervals depending on the category of the cue image, i.e., release during the red-dot interval for more cat-like images and release during the green-dot interval for more dog-like images. The monkey obtained a liquid reward for releasing the bar during the correct interval, and a 4 s timeout for releasing the bar during the incorrect interval. The reward size for release during red-dot and green-dot intervals was 1 and 4 drops, respectively, to compensate for the temporal discounting effect caused by different waiting times. The cue image (10° × 10° visual angle) for each trial was pseudo-randomly selected from a set of 440 morphed images (Figure 1B). The monkey was randomly rewarded in trials with 50% morphed cue images. A black and white noise background prevented afterimages (Figure 1A) when cue images were turned off.

### 2.3 Session selection and trial exclusion

In a prior study Eldridge et al. (2018), all monkeys had been trained to categorize cat/dog images presented for the duration of the trial. In the present study, some monkeys required a period of acclimation to the short-duration presentations before stable performance was obtained. Unless otherwise stated, the analyses presented below included only data collected from sessions after performance reached a stable level, i.e., three successive days above threshold performance. For each monkey, the performance threshold was set to 80% of the highest correct rate, with a floor of 50%.

The reaction time of bar release during the first interval, cued by a red dot (“release-on- red” choice) is defined as the time elapsed from the cue image onset to the bar release, which includes time for visual processing and for motor execution. We observed a long tail in the reaction time distribution (Supplementary Figures S1A–C). For the trials with longer reaction times, we infer that the monkey may not be attending to the task. To separate the reaction times under the “inattentive” condition from those in which the monkey was attending to the task, the reaction times were fitted with a bimodal distribution function, which is a weighted sum of a generalized extreme value (GEV) distribution and a normal distribution (Li et al., 2021). The crossing point of the two distributions was used as the threshold and trials with reaction times longer than the threshold were excluded from subsequent analysis. The threshold, proportion of excluded trials, and the mean and standard deviation of reaction time for each monkey are summarized in Supplementary Table S1.

### 2.4 Data analysis

The categorization performance data was fitted with the function (Equation 1):


(1) $$y=a+\frac{b}{1+e^{cx+d}}$$

where *x* is the morph level (% dog) of the images, *y* is the proportion of dog choices, and *a*, *b*, *c,* and *d* are free parameters. The maximum gradient of fit was represented by *c*.

The processing time was calculated for each monkey in each session as follows (Equation 2):


(2) $$PT=RT\;\mathrm{on}\;red_{ij}-\bar{RT\;\mathrm{on}\;\mathrm{gree}n_{ij}}$$

where PT is the processing time, RT is the reaction time, *i* is the ambiguity level index (inversed morph level for RT on green), and *j* is the image duration index.

A generalized linear mixed-effects model (GLMM) implemented in R, lme4 (version 1.1–35.5) (Bates et al., 2015), was used to analyze the effect of the treatment group (the control, the TE lesion, or the rhinal lesion) and image duration on the accuracy, reaction time, and processing time of visual categorization. When the analysis focused on the effect of the treatment group, the fixed effects were morph level and the treatment group (with interaction terms), and the random effects were monkey identity and session index. The R formula is as follows (Equation 3):


(3) $$\begin{array}{l} \mathrm{data}\sim \mathrm{morph}*\mathrm{factor}\left(\mathrm{group}\right)+\left(1|\mathrm{monkey}\right)+\left(1|\mathrm{session}\right)\\ +\left(0+\mathrm{morph}|\mathrm{monkey}\right)+\left(0+\mathrm{morph}|\mathrm{session}\right) \end{array}$$

When the effect of image duration was considered, the fixed effects were the treatment group, morph level, image duration, and their interactions, and the random effects were monkey identity and session index. The R formula is as follows (Equation 4):


(4) $$\begin{array}{l} \mathrm{data}\sim \mathrm{morph}*\mathrm{duration}*\mathrm{factor}\left(\mathrm{group}\right)+\\ \left(1|\mathrm{monkey}\right)+\left(1|\mathrm{session}\right)+\left(0+\mathrm{morph}|\mathrm{monkey}\right)+\\ \left(0+\mathrm{duration}|\mathrm{monkey}\right)+\left(0+\mathrm{morph}|\mathrm{session}\right)+\\ \left(0+\mathrm{duration}|\mathrm{session}\right) \end{array}$$

The data for accuracy analysis were fitted with a binomial distribution. The reaction time and processing time were assumed to follow a Gamma distribution.

### 2.5 Fitting of a generalized drift-diffusion model

To study the decision-making process among different treatment groups, we fitted monkeys’ performance (accuracy and reaction time) with a generalized drift-diffusion model (GDDM) (Shinn et al., 2020):


$$dx=\mu \left(x,t,\ldots \right)dt+\sigma \left(x,t,\ldots \right)\;dW$$

where “…” represents task conditions and fittable parameters. The decision variable *x* initiates from a starting position, which has a distribution *X_0,_* and reaches decision boundary *B* to terminate the process. A non-decision time, *t_nd_*, is also included in the model.

Model #1 is parameterized by (Equation 5):


$$\mu \left(x,t,\ldots \right)=\{\begin{array}{c} dM,\;t\leq D\\ d_{0}M,\;\;t>D \end{array}$$

$$\sigma \left(x,t,\ldots \right)=1$$

(5) $$X_{0}\left(x,\ldots \right)=\{\begin{array}{c} \frac{1}{2S_{z}+1},-S_{z}\leq x\leq S_{z}\\ 0,\;\mathrm{otherwise} \end{array}$$

$$B\left(t,\ldots \right)=B_{0}e^{-\tau t}$$

$$p_{i}^{*}\left(t\right)=p_{i}\left(t-t_{nd}\right),\;t_{nd}\sim N\left(t_{j},S_{t}^{2}\right)$$

where *B_0_* equals 1, *M* is the morph level, *D* is the duration of image presentation, and *N* is a normal distribution. The six fittable parameters are drift rates *d* and *d_0_*, the parameter for starting position distribution *S_z_*, bound collapse rate *τ*, and mean (*t_j_*) and standard deviation (*S_t_*) of non-decision time. The probability distribution functions can be viewed as a discrete proxy of the continuous probability density.

To test the importance of each parameter, we compared the performance of Model #1 with four other models by computing the Bayesian information criterion (BIC) and log-likelihood (LL). The BIC and LL values of Models #2–5 were normalized to those of Model #1 before statistical testing to control for variation among monkeys, which is caused by different sample sizes.

Model #2 does not include the starting position parameter (Equation 6):


(6) $$X_{0}\left(x,\ldots \right)=\{\begin{array}{c} 1,\;x=0\\ 0,\;\mathrm{otherwise} \end{array}$$

Model #3 does not separate the drift rate between the image-on and image-off epochs (Equation 7):


(7) $$\mu \left(x,t,\ldots \right)=dM$$

Model #4 employed fixed decision bounds instead of collapsing bounds (Equation 8):


(8) $$B\left(t,\ldots \right)=B_{0}$$

Model #5 does not include the starting position parameter, does not separate the drift rate between the image-on and image-off epochs, and employs fixed decision bounds, which leaves three fittable parameters, *d*, *t_j,_* and *S_t_* (Equation 9):


$$\mu \left(x,t,\ldots \right)=dM$$

$$\sigma \left(x,t,\ldots \right)=1$$

(9) $$X_{0}\left(x,\ldots \right)=\{\begin{array}{c} 1,\;x=0\\ 0,\;\mathrm{otherwise} \end{array}$$

$$B\left(t,\ldots \right)=B_{0}$$

$$p_{i}^{*}\left(t\right)=p_{i}\left(t-t_{nd}\right),\;t_{nd}\sim N\left(t_{j},S_{t}^{2}\right)$$

In the two-interval choice task design used here, the decision process should be reflected only in the reaction time of bar release during the first interval (red-dot interval). Thus, we generated a reaction time distribution for correct trials on more dog-like images (bar release during the green-dot interval) by random sampling with replacement from correct trials on more cat-like images (bar release during the red-dot interval) and sample size equal to the original trial number. A reaction time distribution of incorrect trials on more cat-like images (bar release during green-dot interval) was generated by the same method from incorrect trials on more dog-like images (bar release during red-dot interval). This sampling method matches the accuracy of visual categorization and approximates the reaction time distribution of correct and incorrect trials for use in fitting the drift-diffusion model. There is a potential confound in the estimation of reaction time because this method assumes there would be no difference between the distribution of reaction times on more cat-like and more dog-like images. However, this estimation of reaction times is required for fitting the GDDM.

## 3 Results

Three groups of monkeys—an unoperated control group, a group with bilateral TE removals, and a group with bilateral rhinal cortex removals (Figure 1C)—were tested in a two-interval forced-choice categorization task cued by briefly presented images (Figure 1A).

### 3.1 TE lesion impaired the accuracy of visual categorization, whereas rhinal lesion did not

**Figure 2:**
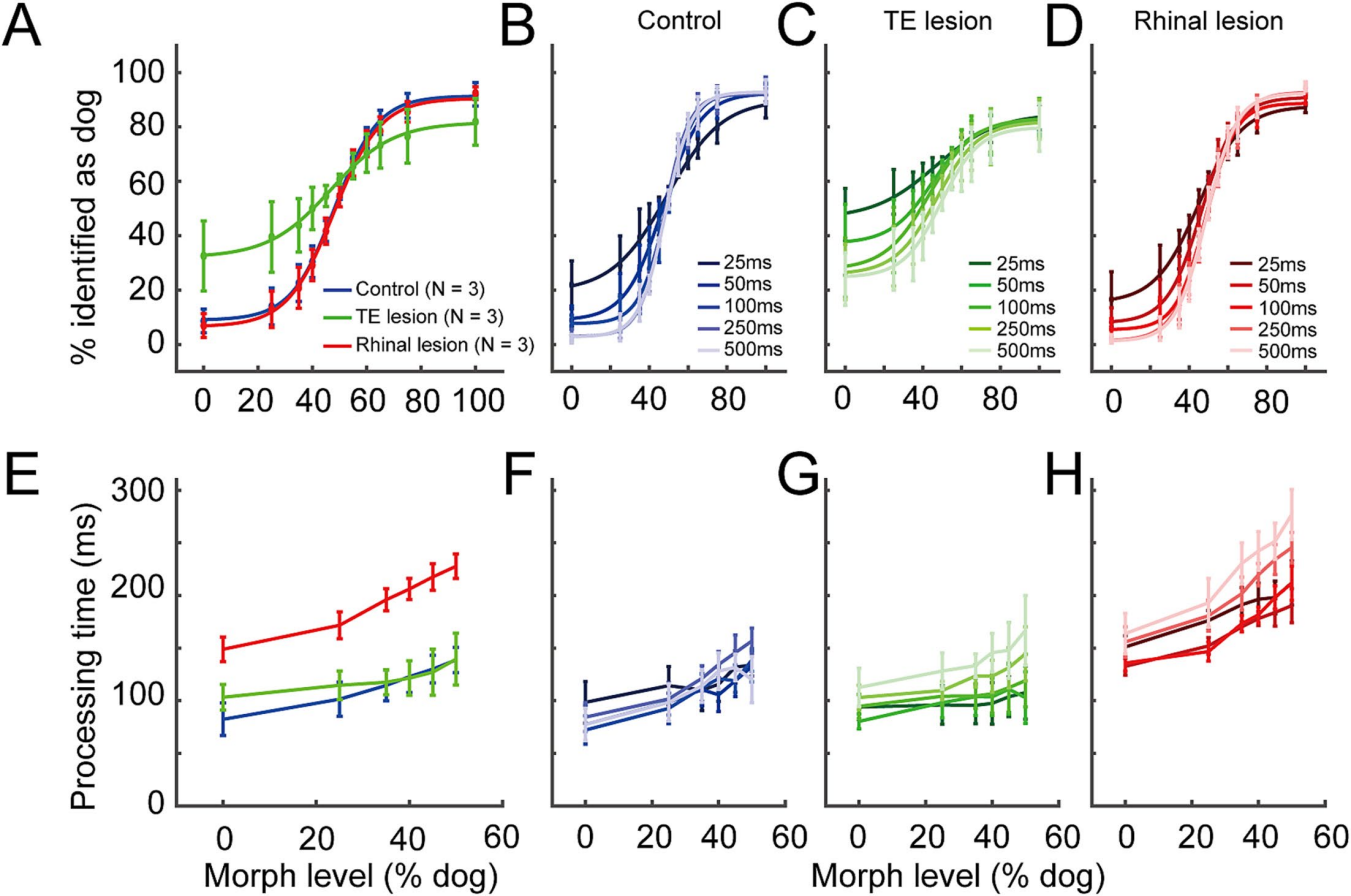
Behavior performance in the control, the TE lesion, and the rhinal lesion groups. **(A)** Accuracy averaged among all image durations in the control (blue line), the TE lesion (green), and the rhinal lesion (red) groups. **(B–D)** Accuracy under different durations of cue image presentation in the control **(B)**, the TE lesion **(C)**, and the rhinal lesion groups **(D)**. Lines in darker to lighter colors represent 25, 50,100, 250, and 500 ms image duration, respectively. **(E)** Processing time averaged among all image durations in the control (blue line), the TE lesion (green), and the rhinal lesion (red) groups. **(F–H)** Processing time under different durations of cue image presentation in the control **(F)**, the TE lesion **(G)**, and the rhinal lesion groups **(H)**. Lines in darker to lighter colors represent 25, 50, 100, 250, and 500 ms image duration, respectively. *n* = 3 monkeys for each group. Error bars represent S.E.M.

The average categorization performance across all image durations (Figure 2A) of the rhinal lesion group (maximum gradient = 11.68, point of subjective equality (PSE) = 48.09% dog) was comparable to that of the control group (maximum gradient = 12.46, PSE = 48.49% dog, GLMM, *p* = 0.78, z = 0.29). In contrast, the psychometric curve of the TE lesion group was flattened (maximum gradient = 8.74), indicating that the accuracy of visual categorization was impaired (GLMM, *p* = 6.05 × 10^−7^, z = 4.99). The curve was also left-shifted (PSE = 40.25% dog), indicating a choice bias. Accuracy of visual categorization was significantly correlated with image duration for all three groups (Figures 2B–D, GLMM, *p* < 2.0 × 10^−16^, z = −16.68).

### 3.2 Rhinal lesion lengthened processing time of categorization, whereas TE lesion did not

In the two-interval task design, the reaction time of bar release during the green-dot interval (“release-on-green”) can be interpreted as the time needed for basic visual-motor processing because the categorization decision has been made before the green dot appears. Indeed, it was not significantly modulated by the task difficulty, i.e., morph level of cue images and image duration (Supplementary Figure S1D, GLMM: morph level, *p* = 0.40, image duration, *p* = 0.62). The visual categorization and decision process is only reflected in the reaction time of “release-on-red” choices. However, the effect of task difficulty and the treatment group on the reaction time for “release-on-red” may not reach the statistically significant level due to the intra-monkey variance in basic visuo-motor processing (Supplementary Figure S1D, GLMM: morph level, *p* = 0.08, image duration, *p* = 0.57, Rhinal lesion, *p* = 0.56, TE lesion, *p* = 0.47). Here, we employed a measure termed “processing time” to reflect the visual categorization and decision process. Processing time was calculated by subtracting the mean reaction time of “release-on-green” choices (representing the basic visuomotor response time) from the reaction time of “release-on-red” choices under each morph level and duration of images (see Methods). Thus, the intra-monkey variance of visuomotor response was removed.

In general, the processing time increased with the increased ambiguity of visual information, i.e., as the morph level of cue images approached 50%, in all the three groups (Figure 2E). Compared to the control group, the averaged processing time across all image durations was significantly lengthened for the rhinal lesion group (GLMM, *p* = 5.62 × 10^−5^, d.f. = 2, *t* = −4.03) (Figure 2E), but that of the TE lesion group was not significantly different from controls (GLMM, *p* = 0.58, d.f. = 2, *t* = −0.55). This suggests that visual categorization/decision-making slowed after the rhinal cortex lesion but not after the TE lesion. Image duration had no consistent effect on the processing time among the three treatment groups (Figures 2F–H, GLMM, *p* = 0.08, d.f. = 4, *t* = 1.73).

### 3.3 GDDM performed better with collapsing decision bounds

**Figure 3:**
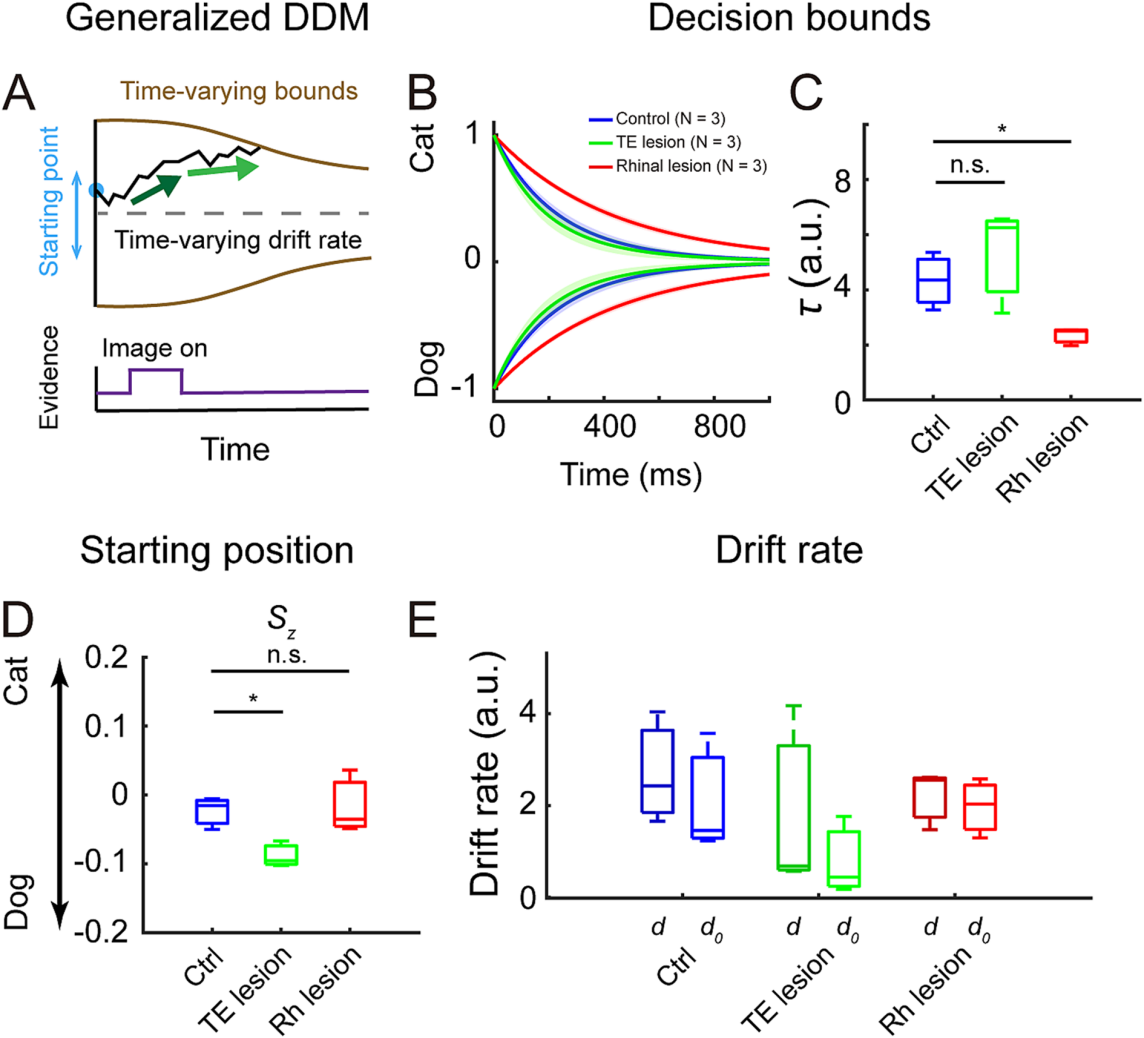
Fitting results of generalized drift-diffusion model (GDDM). **(A)** GDDM: the decision variable starts at a flexible starting point, accumulates in a time-varying drift rate, which is driven by the different strengths of evidence, and reaches a time-varying decision bound to trigger decision. **(B)** Decision bounds collapsed over time in the control (blue), the TE lesion (green), and the rhinal lesion (red) groups. The shaded area represents a 95% confidence interval. **(C)** The collapse rate *τ* of the decision bounds in the three groups. **(D)** The starting position parameter *S_z_* in the three groups. **(E)** Drift rate during image presentation, *d* (darker colors), and after image turned off, *d_0_* (lighter colors), in the three groups.

To study the decision process in more detail, we fitted a generalized drift-diffusion model (GDDM) to the accuracy and reaction time data. We compared the performance between the full model (model #1) and four other models which were constructed by fixing the value of one or more parameters in the full model. All parameters significantly affected the performance of models, but fixing the decision bounds (model #4 and model #5) resulted in poorer fitting than fixing starting position (model #2) or using one drift rate parameter (model #3) (Supplementary Figure S2A, Bayesian information criterion (BIC): model # 1 vs. model #4, *p* = 5.41 × 10^−5^; model # 1 vs. model #5, *p* = 5.41 × 10^−5^; model #1 vs. model #2, *p* = 5.41 × 10^−5^; model #1 vs. model #3, *p* = 3.50 × 10^−3^; log-likelihood (LL): model # 1 vs. model #4, *p* = 5.41 × 10^−5^, model # 1 vs. model #5, *p* = 5.41 × 10^−5^; model #1 vs. model #2, *p* = 5.41 × 10^−5^; model #1 vs. model #3, *p* = 4.92 × 10^−4^, *Kolmogorov–Smirnov* test). Model #1 (Figure 3A) is used for comparing fittable parameters among the three treatment groups as it had the best performance and fully reflected the task design. It also captured the distributions of reaction times in both correct and incorrect trials for the monkeys in all three treatment groups (Supplementary Figure S2B).

### 3.4 Rhinal lesion changed decision bounds and TE lesion changed starting position

Compared to the control group, the bounds collapsed more slowly in the rhinal lesion group but collapsed at a similar rate in the TE lesion group (Figure 3B). The collapse rate *τ* was significantly decreased in the rhinal lesion group compared to controls but was not distinguishable between the TE lesion and the control groups (Figure 3C, control vs. rhinal lesion: *p* = 0.03; control vs. TE lesion: *p* = 0.32; *Kolmogorov–Smirnov* test). These observations suggest that the decision strategy was changed after the rhinal cortex lesion but not after the TE lesion.

The starting position *S_z_* of the decision variable was significantly biased to the dog choice in the TE lesion group compared to the control group (Figure 3D, *p* = 0.03, Kolmogorov–Smirnov test), whereas it was comparable between the rhinal lesion and control groups (*p* = 0.98, Kolmogorov–Smirnov test). There was no significant difference between the drift rate during image presentation (*d*) and the rate after the image disappeared (*d_0_*) in all three treatment groups (Figure 3E, control: *p* = 0.32; TE lesion: *p* = 0.32; rhinal lesion: *p* = 0.98, Kolmogorov–Smirnov test). The drift rate did not change in the TE lesion or rhinal lesion group compared to the control group, neither during image presentation nor after the image disappeared (TE lesion: *p* = 0.32 for *d* and *p* = 0.32 for *d_0_*; rhinal lesion: *p* = 0.98 for *d* and *p* = 0.98 for *d_0_*, Kolmogorov–Smirnov test).

## 4 Discussion

We observed a dissociation between the deficits after bilateral TE removal and those after rhinal removal on accuracy and processing time of visual categorization; TE lesions impaired accuracy but not processing time whereas rhinal lesions did not affect accuracy but lengthened processing time. A generalized drift-diffusion model, fitted to the data, revealed that rhinal lesions slowed the collapse of the decision boundary, whereas TE removal biased the starting position of the decision variable.

### 4.1 Categorization relied on visual input

In the present task, the large number of images (440) and the variety across images (40 different series) make it hard for the monkey to memorize the association with the action, i.e., release on which interval, for each individual image. Monkeys’ performance accuracy decreased as task difficulty increased (Figure 2), which suggests that the monkeys were doing the task by categorization rather than association.

We speculated that following stimuli presented for very short durations, short-term memory may provide category information to the decision-making process after the image disappears. According to this hypothesis, the decision-making time would be lengthened at shorter image durations because category information stored in short-term memory will be weaker than that obtained from visual input, and hence, evidence accumulation will be slowed. However, the processing time of visual categorization in the present task (lower than 300 ms) was much shorter than it was in the continuously cued visual categorization task (500–600 ms) (Eldridge et al., 2018); both tests were performed in the same group of monkeys. Counter to our initial hypothesis, shorter presentation times resulted in shorter processing times (Figures 2G,H). This suggests that the subjects relied primarily on visual input, not short-term memory, to make categorization decisions.

### 4.2 Expediting decisions to maximize reward

We studied the decision-making process by fitting a generalized drift-diffusion model. The introduction of collapsing decision bounds significantly improved the goodness of fit (Supplementary Figure S2A). This indicates that the cumulative evidence required to trigger a decision decreased over time; i.e., choices were taken on the basis of less evidence if evidence accumulation was too slow. Previous studies suggest that collapsing decision bounds may be favored when task difficulty/uncertainty is high (Malhotra et al., 2018), and/or when the subjects are very experienced in the task (Hawkins et al., 2015). Expediting decisions maximize total reward in these conditions (Tajima et al., 2016). In the present task, the shortened image durations increased task difficulty and the interleaved image durations introduced uncertainty. These manipulations may explain why monkeys sped up their decisions relative to our previous, continuously cued, study.

### 4.3 TE lesions impaired perceptual processing but did not alter decision strategy

The accuracy of visual categorization was significantly impaired after bilateral TE lesions (Figure 2A), and decisions were biased toward the “dog” choice (Figures 2A, 3D). The latter indicates that monkeys preferred the choice of offering a bigger reward at a longer delay when the visual evidence was unreliable. The impairment in visual processing after TE lesions is consistent with previous reports (Matsumoto et al., 2016; Eldridge et al., 2018; Setogawa et al., 2021). The effect of image duration on visual categorization accuracy was comparable between the TE lesion group and controls (Figures 2B,C), indicating that the visual categorization function was not entirely abolished after the TE lesion.

Processing time was unaffected by TE lesions (Figures 2E–G) as was the rate of decision-bound collapse (Figures 3B,C). These results suggest that the decision strategy was unchanged after the TE lesion.

### 4.4 Rhinal lesions slowed decision-making but did not impair perceptual processing

After bilateral rhinal lesions the accuracy of visual categorization was intact (Figures 2A,B,D). This suggests that the rhinal cortex did not contribute to the perceptual processing of these stimuli, which is consistent with our earlier study (Eldridge et al., 2018). Previous studies have suggested that the rhinal cortex might play an important role in object recognition when short-term memory is required (Meunier et al., 1993; Buffalo et al., 1999). However, the visual categorization in this task placed demands primarily on visual input, not short-term memory (as discussed above in the section “Categorization relied on visual input”).

Processing time was significantly lengthened in the rhinal lesion group compared to the control group (Figures 2E,F,H). The fitting results showed that the decision bounds in the rhinal lesion group collapsed more slowly than those of the control group (Figures 3B,C). As mentioned above, the shortened image duration may be the driving force for faster decisions in the present task, i.e., the likelihood of gathering reliable category information decreases over time. Monkeys may develop a prediction of image duration—a “decision deadline”—to optimize the collection of category information. Lesions of the rhinal cortex, including the entorhinal cortex (Figure 1C), may impair the prediction of image duration (Tsao et al., 2022; Dias et al., 2021; Heys et al., 2020; Bright et al., 2020; Montchal et al., 2019; Heys and Dombeck, 2018) and, hence, lengthen the processing time. Other possibilities are that efficient decision completion was impaired, rhinal lesion monkeys could have lost sensitivity to the uncertainty of the task—uncertainty may lead to faster decisions (Malhotra et al., 2018)—or motivation to maximize the reward rate was compromised. Such effects could be caused by lesions to the other component of the rhinal cortex, the perirhinal cortex (Figure 1C), which has connections with the orbitofrontal cortex, cingulate cortex, and amygdala (Suzuki and Naya, 2014; Suzuki and Amaral, 1994; Stefanacci et al., 1996; de Curtis and Paré, 2004; Suzuki, 2009). There is evidence from prior studies that attention may be impaired after perirhinal cortex lesions in humans (Barense Morgan et al., 2012). However, impairment in attention would be expected to slow evidence accumulation and lead to longer reaction times in the task we used, but the unchanged drift rate after the rhinal cortex lesion (Figure 3E) does not support this interpretation.

### 4.5 Different roles for area TE and rhinal cortex in visual categorization

We report a double dissociation of the effects of lesions of area TE and lesions of the rhinal cortex in a test of rapid visual categorization. The TE lesion group appeared to seek to maximize their reward rate by maintaining decision-making rates at the same level as controls, despite a significant reduction in categorization accuracy. Rhinal lesions slowed decision-making; possible explanations for this observation are as follows: impaired time prediction, e.g., cannot effectively implement a “decision deadline”; loss of sensitivity to the task difficulty; and reduction in motivation to maximize the reward rate. In conclusion, area TE processes the visual perceptual information that is fundamental for all forms of visual categorization (Cauchoix et al., 2016; Freedman et al., 2003; Meyers et al., 2008), whereas the rhinal cortex supports the type of rapid categorization likely to be important for evaluating the dynamic visual input experienced during natural behavior.

## Data Availability

The datasets presented in this study can be found in online repositories. The names of the repository/repositories and accession number(s) can be found below: figshare (DOI: https://doi.org/10.6084/m9.figshare.26426485).
