# Supplementary material for: Dissociation between area TE and rhinal cortex in accuracy vs. speed of visual categorization in rhesus monkeys
Source: Front Behav Neurosci. 2024 Nov 21;18:1481478. doi: 10.3389/fnbeh.2024.1481478 (PMC11617191; doi:10.3389/fnbeh.2024.1481478)
Supplement: Supplementary file 1 [file Data_Sheet_1.docx]

**Supplementary Table 1** Parameters of trial exclusion

| Group | Monkey | Threshold (ms) | Trial excluded (%) | RT before exclusion (ms) | RT after exclusion (ms) |
| --- | --- | --- | --- | --- | --- |
| Control | P | 741.00 | 4.99 | 524.18±204.11 | 486.32±80.19 |
|  | Te | 799.00 | 2.15 | 455.40±117.53 | 441.47±75.69 |
|  | E | 869.30 | 3.09 | 523.06±181.32 | 499.08±100.40 |
| TE-lesion | T | 979.9 | 1.54 | 457.03±186.14 | 434.64±115.88 |
|  | K | 1085.43 | 1.07 | 476.38±404.30 | 422.83±111.31 |
|  | G | 648.00 | 4.98 | 435.63±145.58 | 411.90±75.54 |
| Rhinal-lesion | M | 1055.28 | 1.64 | 574.95±168.43 | 558.87±123.71 |
|  | C | 904.52 | 3.81 | 542.54±210.34 | 506.13±121.02 |
|  | B | 1175.80 | 2.51 | 558.85± 285.69 | 514.43±136.26 |

RT: reaction time

**Supplementary Table 2** BIC of Generalized drift-diffusion model (GDDM) fitting

| Group | Monkey | Model  #1 | Model  #2 | Model  #3 | Model  #4 | Model  #5 | Sample  size |
| --- | --- | --- | --- | --- | --- | --- | --- |
| Control | P | -29984 | -29809 | -29782 | 27679 | 26053 | 26054 |
|  | Te | -14315 | -14315 | -14296 | -11211 | -23009 | 7364 |
|  | E | -10326 | -10162 | 10294 | 19871 | 19815 | 14951 |
| TE-lesion | T | -19500 | -18585 | -17971 | 23004 | 28872 | 25265 |
|  | K | -37731 | -35381 | -37756 | 40287 | 41418 | 13594 |
|  | G | -27808 | -26090 | -27807 | 62839 | 74463 | 25104 |
| Rhinal-lesion | M | -80783 | -79223 | -80564 | 34214 | 33266 | 25730 |
|  | C | -19802 | -19643 | -19826 | 12317 | 11156 | 26234 |
|  | B | -15176 | -15017 | -15075 | 21148 | 19459 | 28240 |
| Average |  | -16529  ±8712 | -16120  ±8447 | -16320  ±8630 | 26693±18097 | 28022±21543 |  |

**
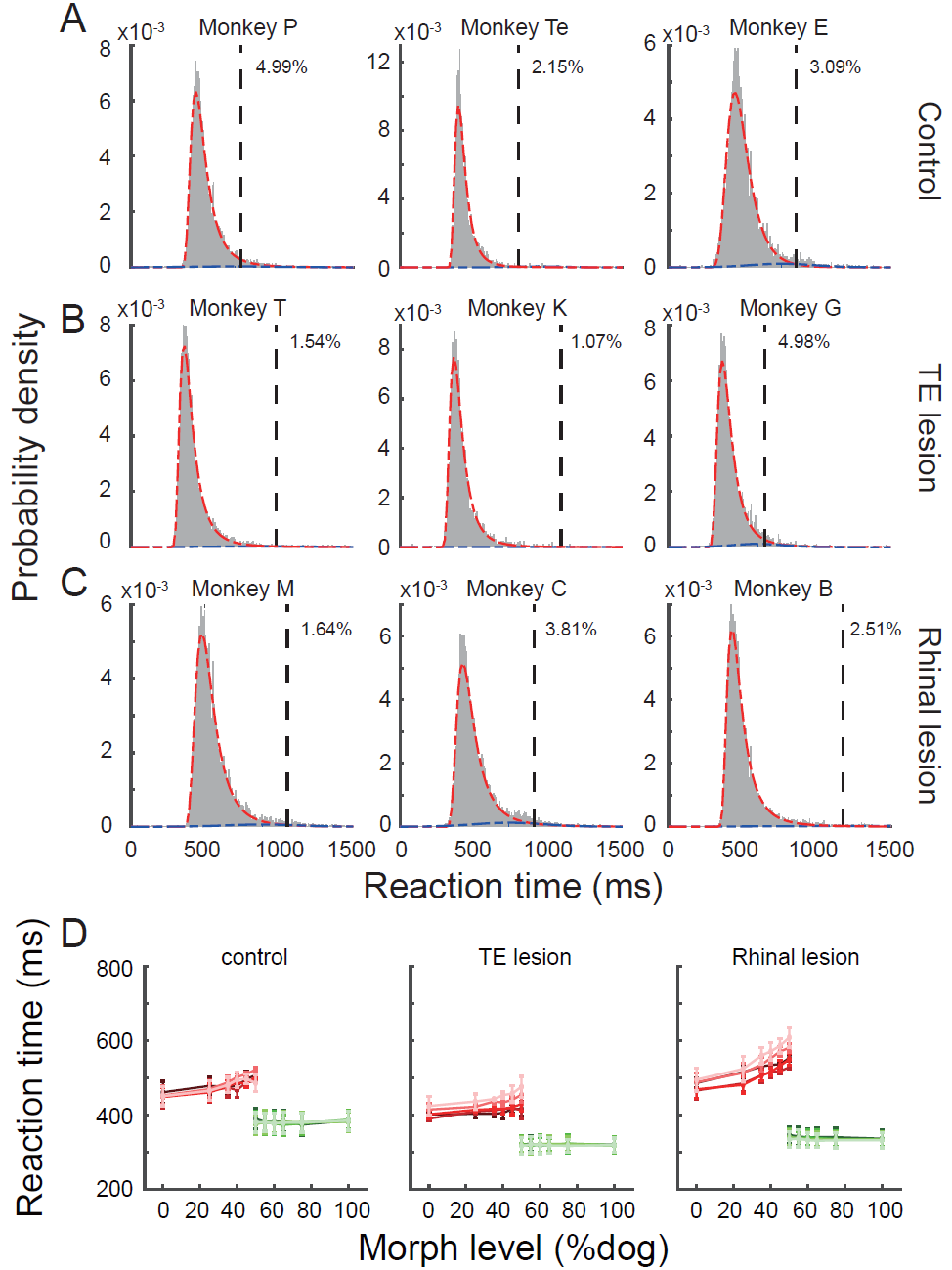
**

**Supplementary Figure 1.** Reaction time in control, TE lesion and rhinal lesion groups. Reaction time distribution of each monkey in control (A), TE lesion (B) and rhinal lesion (C) groups. Red and blue dashed lines represent the bimodal fitting of the reaction time distribution. Black dashed line represents the threshold for trial exclusion. (D) Red lines represent the reaction time of bar release during red-dot interval in response to the more cat-like images. Green lines represent the reaction time of bar release during green-dot interval in response to the more dog-like images. Darker to lighter color represents the reaction time under 25, 50, 100, 250, 500ms image durations. Error bars represent S.E.M.

**
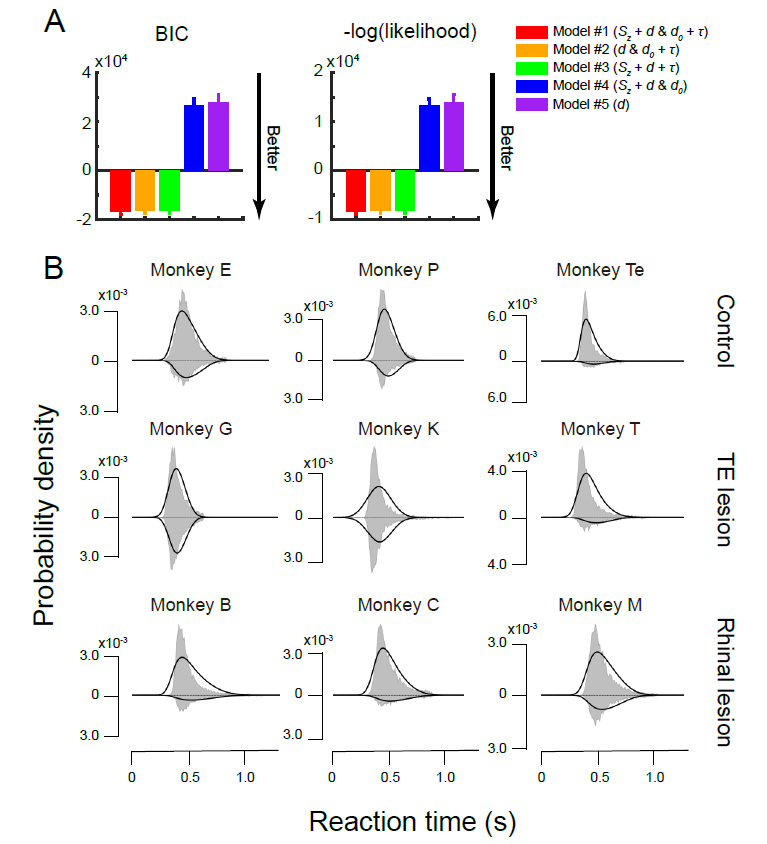
**

**Supplementary Figure 2.** Model selection and fitting results. (A) BIC and loglikelihood comparison among five models. Error bars represent S.E.M. (B) Probability density of reaction time of the experimental data (shaded area) and the model (black line). The upper and lower distribution represents the reaction time of correct and wrong trials, respectively.
